# NADH-based kinetic model for acetone-butanol-ethanol production by *Clostridium*

**DOI:** 10.3389/fbioe.2023.1294355

**Published:** 2023-11-24

**Authors:** Juan Carlos Quintero-Díaz, Diego F. Mendoza, Claudio Avignone-Rossa

**Affiliations:** ^1^ Department of Chemical Engineering, Universidad de Antioquia, Medellín, Colombia; ^2^ Department of Microbial Sciences, School of Biosciences, University of Surrey, Guildford, United Kingdom

**Keywords:** redox mediator, ABE fermentation, mathematical structured modeling, parametric sensitivity, electrofermentation

## Abstract

We present in this work a kinetic model of the acetone-butanol-ethanol (ABE) fermentation based on enzyme kinetics expressions. The model includes the effect of the co-substrate NADH as a modulating factor of cellular metabolism. The simulations obtained with the model showed an adequate fit to the experimental data reported by several authors, matching or improving the results observed with previous models. In addition, this model does not require artificial mathematical strategies such as on-off functions to achieve a satisfactory fit of the ABE fermentation dynamics. The parametric sensitivity allowed to identify the direct glucose → acetyl-CoA → butyryl-CoA pathway as being more significant for butanol production than the acid re-assimilation pathway. Likewise, model simulations showed that the increase in NADH, due to glucose concentration, favors butanol production and selectivity, finding a maximum selectivity of 3.6, at NADH concentrations above 55 mM and glucose concentration of 126 mM. The introduction of NADH in the model would allow its use for the analysis of electrofermentation processes with *Clostridium*, since the model establishes a basis for representing changes in the intracellular redox potential from extracellular variables.

## 1 Introduction

Butanol has become an attractive renewable energy source for use in internal combustion engines, due to presenting properties similar to those of gasoline (Li et al., 2019; Re and Mazzoli, 2023). It also has advantages over alcohols such as ethanol in that it has a lower auto-ignition temperature, is less corrosive, has lower volatility and higher energy per unit mass. Furthermore, butanol can be blended with gasoline in high proportions and even replace gasoline, while ethanol is used as an additive (Li et al., 2019; Pfromm et al., 2010; dos Santos et al., 2022).

Both ethanol and butanol can be produced from agro-industrial residues of potato, carrot, onion, banana, sugar cane, coffee, among others (Singh et al., 2021; Mujtaba et al., 2023); however, butanol cannot compete commercially with ethanol, due to the lower yield and productivity of the fermentation process (Pfromm et al., 2010). In spite of this, the global bio-butanol market was estimated at 3 billion gallons in 2020 and an annual growth rate of more than 6.8% is expected between 2022 and 2028 (Zabed et al., 2023), Therefore, optimization of the fermentation process is necessary to achieve greater competitiveness against ethanol.

**FIGURE 1:**
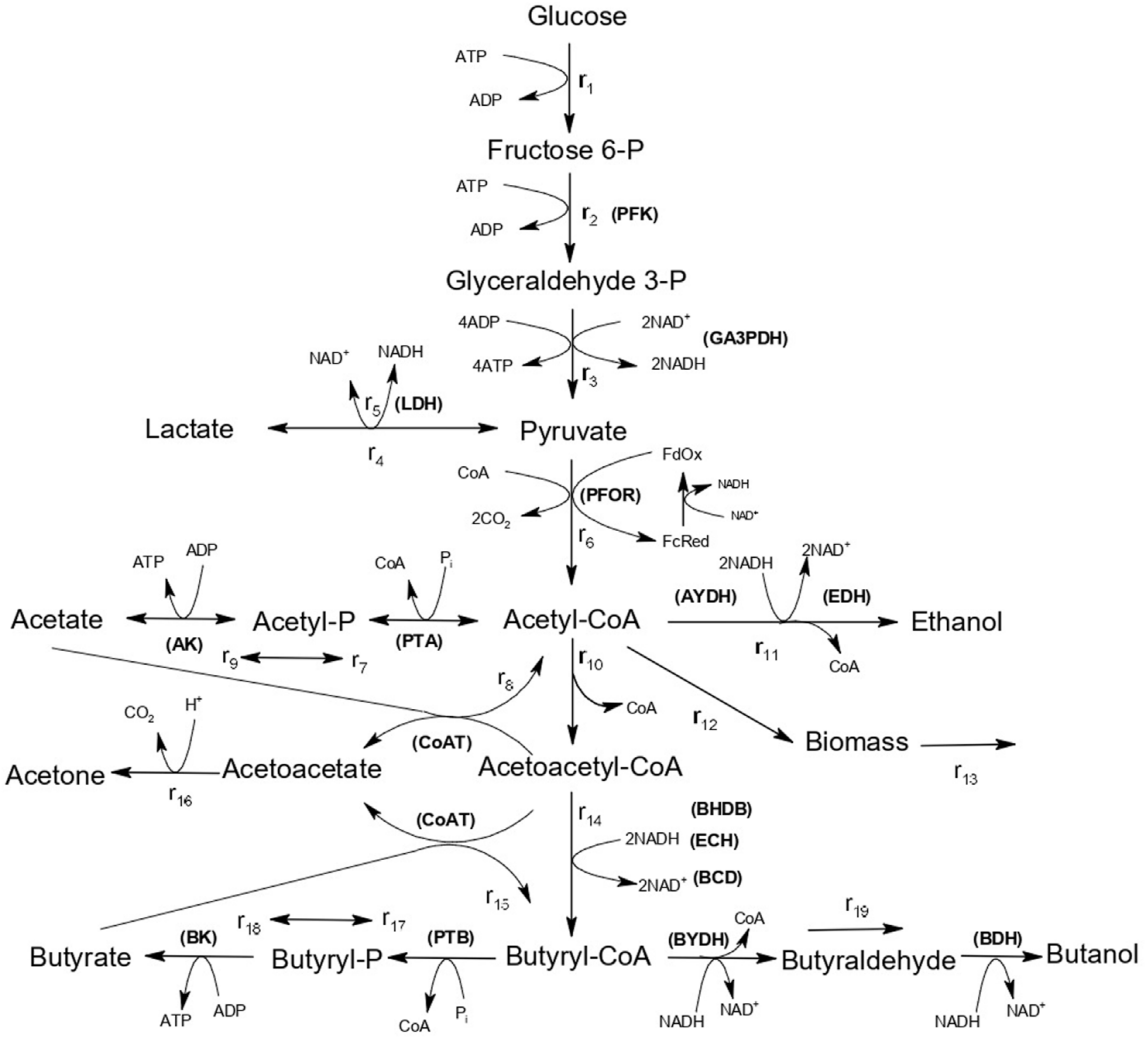
Pathways of ABE metabolism in *C. saccharoperbutylacetonicum N1-4*. Enzymes are indicated in capital letters and abbreviated as follows. PFK: 6-Phosphofructokinase; DA3PDH: Glyceraldehyde-3-phosphate dehydrogenase; LDH: Lactate dehydrogenase; PFOR: Pyruvate-ferrodoxin oxidoreductase; AK: Acetate kinase; PTA: Phosphotransacetylase; AYDH: Acetaldehyde dehydrogenase; ADH: Ethanol dehydrogenase; CoAT: Acetoacetyl-CoA:acetate/butyrate:CoA-transferase; BHBD: *β*-hydroxybutyryl-CoA-dehydrogenase; ECH: Enoyl-CoA hydratase; BCD: Butyryl-CoA dehydrogenase; BK: Butyrate kinase; PTB: Phosphate butyryltransferase; BYDH: Butyraldehyde dehydrogenase; BDH: Butanol dehydrogenase.

The production of butanol through fermentation is carried out by bacterial species belonging to the genus *Clostridium*, which are strict anaerobes. The process is known as ABE fermentation because the main products of fermentation are Acetone, Butanol and Ethanol, with a typical ratio of 3:6:1, respectively (Niglio et al., 2019). The metabolism of ABE fermentation consists of two phases. During the first phase (acidogenic phase), sugars are converted into butyric and acetic acids (with butyric acid being the predominant one), resulting in a decrease in pH. In the second phase (solventogenic phase), the acids act as cosubstrates and are re-assimilated for the production of butanol as the main product, with acetone and ethanol as minor byproducts (Figure 1). The low yields of ABE fermentation are associated with substrate (glucose) inhibition (Zabed et al., 2023), product (butanol) inhibition (Bowles and Ellefson, 1985), and the presence of acids primarily in their undissociated state (Van Ginkel and Logan, 2005).

Efforts have been made to understand the metabolic mechanisms associated with the pH change that accompanies the transition from the acidogenic phase to the solventogenic phase. However, it is still unclear how this transition is controlled, and it is generally accepted that the accumulation of acetic and butyric acids, which leads to a decrease in pH, triggers the phase transition (Wang et al., 2011; Al-Shorgani et al., 2018).

A mathematical analysis of the biochemical phenomena involved in biological processes provides an important guide for conducting metabolic engineering studies and for the design and optimization of fermentation systems (Almquist et al., 2014; Strutz et al., 2019). Mathematical models allow the description of those phenomena through kinetic expressions, mass balance equations, and transport equations, facilitating the simulation of fermentation processes. Simulation processes help reduce the extensive experimental work that would be required to analyze different physical, design and process variables, and they help identify the influence of those variables on the process and their interdependence (Liao et al., 2016; Millat and Winzer, 2017).

ABE fermentation processes have been described using phenomenological mathematical models based on the metabolism shown in Figure 1. Some of these models incorporate slight modifications to certain reactions while maintaining a consistent structure in the description of kinetic equations based on the Michaelis-Menten model. Due to the lack of knowledge regarding the metabolic mechanisms associated with pH changes, it has become common in a significant number of studies to describe the behavior of ABE fermentation using on-off functions that artificially capture phenomena such as substrate inhibition, product (butanol) inhibition, or the effect of pH to describe the transition from the acidogenic phase to the solventogenic phase.

Shinto et al. (2008), Shinto et al. (2007) proposed a mathematical model to describe ABE fermentation of *C. saccharoperbutylacetonicum* N1-4, based on the metabolism depicted in Figure 1. This model consists of 19 kinetic equations, 16 metabolites, and 45 parameters, and includes an on-off mechanism that sets reaction rates to zero when the glucose concentration in the medium is below 1.0 mM. Recent works based on this model still employ the artificial on-off mechanism (Raganati et al., 2015; Díaz and Tost, 2016; Díaz and Willis, 2018).

Buehler and Mesbah (2016) proposed a model to describe ABE fermentation of *Clostridium acetobutylicum* in continuous culture, also based on the metabolism depicted in Figure 1. In this case, biomass production from glucose was considered. The model incorporates two on-off mechanisms. The first mechanism, equivalent to the one proposed by Shinto et al. (2008), Shinto et al. (2007), simulates the suppression of cellular metabolism at glucose concentrations below 2.0 mM. The second on-off mechanism includes an inhibition function of biomass growth for pH values below 5.6. Another strategy employed in mathematical models is the replacement of artificial on-off functions by functions that describe the enzymatic activity kinetics associated with some of the metabolic reactions. In this case, step-like on-off functions are used to maintain constant profiles of enzyme activity during specific time intervals throughout the fermentation process (Li et al., 2011).

Other approaches to the mathematical description of ABE fermentation include the utilization of unstructured models, which mitigate the complexities linked to the mathematical representation of metabolic pathways (Velázquez-Sánchez and Aguilar-López, 2018; Li et al., 2019; Rivas-Astroza et al., 2021). More recently, kinetic models based on cell-free systems have also emerged as an alternative methodology (Karim and Jewett, 2016; Martin et al., 2023).

From a biochemical perspective, it has been considered that the metabolism of pyruvate appears to be the key trigger for the conversion of acids into solvents, and the reactions involved primarily relate to electron transfer between the oxidized and reduced forms of NADH (Kim et al., 1988). Furthermore, it has been demonstrated that the levels of NADH/NAD^+^ in *C. acetobutylicum* are closely related to solvent production (Wang et al., 2012; Karim et al., 2018). These pieces of evidence have led to the possibility of making environmental modifications in the extracellular redox potential in *Clostridium* cultures to modify the intracellular redox potential through the availability of NADH (Ou et al., 2015; Mogollón et al., 2023).

Recently, a kinetic model has been presented for ABE fermentation, which represents an initial attempt to incorporate NADH into the kinetic description of metabolism. In this case, the modulating effect of the NADH/NAD ratio on Michaelis-Menten kinetics was included (Chalhoub et al., 2023). Nevertheless, this ratio remains constant during fermentation, and NADH is not included as a co-substrate in the kinetic models. Several studies have shown that the NADH/NAD ratio varies significantly during fermentation, as does the concentration of NADH (Wang et al., 2011; He et al., 2016; Zhou et al., 2023). Therefore, taking into account the dynamic effect of this co-substrate on the kinetics of fermentation could be significant. Possibly due to not taking these considerations into account, the adjustments of this latest model to the experimental data were poor (Chalhoub et al., 2023).

It can be observed in Figure 1, that reactions *r*_11_, *r*_14_, and *r*_19_ involve the consumption of NADH, which acts as a co-substrate for the enzymes acetaldehyde dehydrogenase (AYDH) (*r*_11_), beta-hydroxybutyryl-CoA dehydrogenase (BHBD) (*r*_14_), and butyraldehyde dehydrogenase (BYDH) (*r*_19_). Therefore, this work proposes to include the effect of the presence of the cosubstrate NADH as a modulating factor in the kinetic models. This, in turn, allows for the elimination of artificial on-off functions to achieve a satisfactory description of ABE fermentation.

We present here a model for butanol fermentation based on the central ABE metabolism of *Clostridium*. The main contributions of this paper are:

• The inclusion of the redox mediator NADH as metabolic regulator in the ABE fermentation of *Clostridium.*
• The use of first-principles (biochemical-based) kinetic expressions to represent the reactions in the model.
• The avoidance of artificial on-off variables to represent the metabolic regulation during the ABE fermentation.

The model adequately represents the experimental results reported by various authors. Further, a parametric sensitivity analysis was conducted to highlight the impact of metabolic reactions on solvent production. Simulations performed using the model allowed the evaluation of different fermentation scenarios and revealed the influence of NADH on ABE solvent production.

## 2 Materials and methods

### 2.1 Construction of a model for ABE metabolism

The proposed mathematical model is based on the metabolic model presented in Figure 1, describing glucose fermentation by *C. acetobutylicum* (Gheshlaghi et al., 2009). Glucose is converted to pyruvate via the glycolytic pathway, and pyruvate is predominantly converted to acetyl-CoA by the enzyme pyruvate–ferredoxin (Fd) oxidoreductase (PFOR). Acetyl-CoA is a central node in the metabolic pathway, from which all the relevant products (acetate, ethanol, butanol and butyrate) are formed by the activity of one or more enzymes. During the initial acidogenic phase, acetic and butyric acids, along with ATP, are produced, which is associated to cell growth. Subsequently, a portion of the acids is re-assimilated for solvent production during the solventogenic phase (Jang et al., 2012a). It has been proposed that butanol can also be produced directly from acetyl-CoA to butyryl-CoA without the re-assimilation of acids (Jang et al., 2012b). This dual metabolism model involves butanol production via a direct route (the “*hot channel*”) and a re-assimilation route (the “*cold channel*”) (Jang et al., 2012b).

The conversion of 2 mol of glucose to 2 mol of acetyl-CoA is accompanied by the production of 2 mol of NADH from NAD^+^ in the glycolytic pathway. NAD^+^ is regenerated to achieve intracellular redox balance necessary for glycolysis to continue.

ABE metabolism presents three stages that result in NAD^+^ regeneration (Figure 1): 1) ethanol production via AYDH and EDH; 2) butanol production via BYDH and BDH; and 3) the formation of butyryl-CoA via BHBD and BCD from acetoacetyl-CoA. This is associated with the high dependency of the enzymes AYDH, EDH, BYDH, and BHBD on NADH (Gheshlaghi et al., 2009; Yoo et al., 2015). Therefore, including the NADH dependency in the kinetic models of enzyme kinetics allows for the development of a mathematical model that describes the modulating function of NADH on ABE fermentation.

### 2.2 Model development-kinetic equations

The mathematical model used in this study is based on the model proposed for ABE production by *C. acetobutylicum* (Shinto et al., 2007), described by the metabolic network shown in Figure 1. It consists of 19 reaction rate equations, 16 intracellular and extracellular mass balance equations, and 47 parameters.

The kinetics of reactions *r*_2_ to *r*_7_, *r*_9_, *r*_10_, *r*_16_, and *r*_18_ are described using the enzyme-substrate reaction mechanism for single-substrate systems, Eq. 1, from which the Michaelis-Menten rate equation (Eq. (2)) is derived (Wang and Post, 2013).

$$E+S\overset{k_{1}}{\underset{k_{-1}}{⇌}}ES\overset{k_{2}}{\to }E+P$$ (1)

$$r_{i}=r_{i\max}\;\frac{\left[S\right]}{K_{M}+\left[S\right]}\;$$ (2)

Where [*S*] is the substrate concentration (mM), *K*_*m*_ is the Michaelis-Menten constant (mM), *r*_*i*_ is the reaction rate of component *i* (h^−1^), and *r*_*i*__*max*_ is the maximum reaction rate (h^−1^).

The rates *r*_8_ and *r*_15_ corresponding to the re-assimilation of acetic and butyric acids, respectively, to acetoacetyl CoA, are described using Michaelis-Menten kinetics, considering the effect of these two substrates individually. The cell growth rate *r*_12_ is also described using Michaelis-Menten kinetics.

While glucose is the most common substrate (Buehler and Mesbah, 2016; Velázquez-Sánchez and Aguilar-López, 2018), other metabolites have been proposed to account for the substrate dependence on the growth rate, including acetyl-CoA (Shinto et al., 2007; Li et al., 2011) and butyryl-CoA (Díaz and Willis, 2018). In this work, acetyl-CoA was considered to be the limiting substrate, and the inhibitory effect of butanol on the cell growth rate was included (Soni et al., 1987), since acetate, butyrate, acetone, and ethanol rarely reach toxic levels to inhibit biomass growth (Bowles and Ellefson, 1985; Chen et al., 2012; Buehler and Mesbah, 2016). Cell death rate (*r*_13_) was assumed to be a first-order function of the biomass concentration. Glucose consumption rate (*r*_1_) was also represented by Michaelis-Menten kinetics, considering the inhibitory effect of the substrate (glucose) and the inhibitory effect of butanol (Chen et al., 2012; Zabed et al., 2023). Furthermore, the Michaelis-Menten model for butyryl-CoA production (*r*_17_) included the inhibitory effect of butyrate (Gheshlaghi et al., 2009).

The production of hydrogen has not been considered in the model because the [FeFe]-hydrogenase activity in *C. acetobutylicum* is not detectable at the pH levels lower than 6.0 observed in ABE fermentations (Khanal et al., 2004; Gheshlaghi et al., 2009; Morra et al., 2016; Mogollón et al., 2023).

To account for the effect of reducing power on the modulation of ABE metabolism, NADH was included as a co-substrate in the kinetic equations describing the rate of reactions *r*_11_, *r*_14_, and *r*_19_, which are the steps where NADH is consumed to regenerate NAD^+^. This consideration, to the best of our knowledge, has not been previously addressed by other models.

The interaction of the enzyme with the substrate and co-substrate in these reactions follows a ping-pong enzymatic mechanism (Yan and Chen, 1990; Gheshlaghi et al., 2009), which is described by Eqs. 3, 4. The corresponding kinetic model for this mechanism is presented in Eq. 5.

$$E+S1\overset{k_{1}}{\underset{k_{-1}}{⇌}}ES1⇌ES1+S2\overset{k_{2}}{\underset{k_{-2}}{⇌}}ES1S2$$ (3)

$$ES1S2\overset{k_{3}}{\underset{k_{-3}}{⇌}}EP1P2⇌EP2+P1\overset{k_{4}}{\underset{k_{-4}}{⇌}}E+P2$$ (4)

$$r_{i}=r_{i\max}\;\frac{\left[S_{1}\right]\;\left[S_{2}\right]}{K_{M1}\;\left[S_{1}\right]+K_{M2}\;\left[S_{2}\right]+\left[S_{1}\right]\;\left[S_{2}\right]}$$ (5)

Where [*S*_1_] and [*S*_2_] are the concentrations of substrate and cosubstrate (mM), *r*_*i*__*max*_ is the maximum reaction rate (h^−1^) achieved at the limit of infinite concentrations of [*S*_1_] and [*S*_2_], *K*_*M*1_ and *K*_*M*2_ are the Michaelis-Menten constants (mM) for [*S*_1_] and [*S*_2_], respectively.

On the other hand, Eq. 6 corresponds to the kinetics of the ping-pong mechanism with the effect of a competitive inhibitor, where the term [*S*_1_] [*S*_2_] in the denominator is multiplied by (1 + [*I*]/*K*_*i*_), where [*I*] is the concentration of the inhibitor and *K*_*i*_ is the dissociation constant of the enzyme-inhibitor complex.

$$r_{i}=r_{i\max}\;\frac{\left[S_{1}\right]\;\left[S_{2}\right]}{K_{M1}\;\left[S_{1}\right]+K_{M2}\;\left[S_{2}\right]+\left[S_{1}\right]\;\left[S_{2}\right]\;\left(1+\left[I\right]/K_{i}\right)}$$ (6)

This kinetic model represents the inhibitory effect of butanol in reaction *r*_19_ (Gheshlaghi et al., 2009). The kinetic models are described by Eqs. 7–25.

$$r_{1}=V_{1}\;\frac{\left[G\right]}{K_{1}\left(1+\frac{\left[G\right]}{K_{1A}}\right)+\left[G\right]\left(1+\frac{\left[ButOH\right]}{K_{1B}}\right)}$$ (7)

$$r_{2}=V_{2}\;\frac{\left[F6P\right]}{K_{2A}+\left[F6P\right]}$$ (8)

$$r_{3}=V_{3}\;\frac{\left[G3P\right]}{K_{3A}+\left[G3P\right]}$$ (9)

$$r_{4}=V_{4}\;\frac{\left[Lac\right]}{K_{4A}+\left[Lac\right]}$$ (10)

$$r_{5}=V_{5}\;\frac{\left[Pyr\right]}{K_{5A}+\left[Pyr\right]}$$ (11)

$$r_{6}=V_{6}\;\frac{\left[Pyr\right]}{K_{6A}+\left[Pyr\right]}$$ (12)

$$r_{7}=V_{7}\;\frac{\left[Ac\right]}{K_{7A}+\left[Ac\right]}$$ (13)

$$r_{8}=V_{8}\;\frac{\left[Ac\right]}{K_{8A}+\left[Ac\right]}\;\cdot \;\frac{\left[AACoA\right]}{K_{8B}+\left[AACoA\right]}$$ (14)

$$r_{9}=V_{9}\;\frac{\left[ACoA\right]}{K_{9A}+\left[ACoA\right]}$$ (15)

$$r_{10}=V_{10}\;\frac{\left[ACoA\right]}{K_{\mathrm{10A}}+\left[ACoA\right]}$$ (16)

$$r_{11}=V_{11}\;\frac{\left[ACoA\right]\cdot \left[NADH\right]}{\left[ACoA\right]\cdot \left[NADH\right]+K_{\mathrm{11A}}\;\left[NADH\right]+K_{\mathrm{11B}}\;\left[ACoA\right]}$$ (17)

$$r_{12}=V_{12}\;\frac{\left[ACoA\right]}{\left(K_{\mathrm{12A}}+\left[ACoA\right]\right)\;\left(1+\frac{\left[BuOH\right]}{K_{\mathrm{12B}}}\right)}$$ (18)

$$r_{13}=K_{\mathrm{13A}}$$ (19)

$$r_{14}=V_{14}\;\frac{\left[AACoA\right]\cdot \left[NADH\right]}{\left[AACoA\right]\cdot \left[NADH\right]+K_{\mathrm{14A}}\cdot \left[NADH\right]+K_{\mathrm{14B}}\cdot \left[AACoA\right]}$$ (20)

$$r_{15}=V_{15}\;\frac{\left[Buty\right]}{K_{\mathrm{15A}}+\left[Buty\right]}\cdot \frac{\left[AACoA\right]}{K_{\mathrm{15B}}+\left[AACoA\right]}$$ (21)

$$r_{16}=V_{16}\;\frac{\left[AcAc\right]}{K_{\mathrm{16A}}+\left[AcAc\right]}$$ (22)

$$r_{17}=V_{17}\;\frac{\left[Buty\right]}{K_{\mathrm{17A}}\left(1+\frac{K_{\mathrm{17B}}}{\left[Buty\right]}\right)+\left[Buty\right]}$$ (23)

$$r_{18}=V_{18}\;\frac{\left[BCoA\right]}{K_{\mathrm{18A}}+\left[BCoA\right]}$$ (24)

$$r_{19}=V_{19}\;\frac{\left[BCoA\right]\cdot \left[NADH\right]}{K_{\mathrm{19A}}\cdot \left[BCoA\right]+K_{\mathrm{19B}}\cdot \left[NADH\right]+\left[BCoA\right]\cdot \left[NADH\right]\left(1+\frac{\left[ButOH\right]}{K_{\mathrm{19C}}}\right)}$$ (25)

### 2.3 Model development-balance equations

The balance equations are given by expressions of the form:

$$\frac{dC}{dt}=v\;r\;X$$ (26)

Where **C** is the vector of the 16 balanced components shown in Figure 1, **v** is the stoichiometric matrix, **r** is the vector of the kinetic equations shown in Eqs. 7–25, and *X* corresponds to the biomass concentration.

$$\frac{d}{dt}\left(\begin{array}{c} \left[G\right]\\ \left[F6P\right]\\ \left[G3P\right]\\ \left[Pyr\right]\\ \left[Lac\right]\\ \left[ACoA\right]\\ \left[X\right]\\ \left[Ac\right]\\ \left[EtOH\right]\\ \left[AACoA\right]\\ \left[AcAc\right]\\ \left[BCoA\right]\\ \left[Buty\right]\\ \left[An\right]\\ \left[BuOH\right]\\ \left[NADH\right] \end{array}\right)=\left(\begin{array}{ccccccccccccccccccc} -1 & 0 & 0 & 0 & 0 & 0 & 0 & 0 & 0 & 0 & 0 & 0 & 1 & 0 & 0 & 0 & 0 & 0 & 0\\ 1 & -1 & 0 & 0 & 0 & 0 & 0 & 0 & 0 & 0 & 0 & 0 & 0 & 0 & 0 & 0 & 0 & 0 & 0\\ 0 & 1 & -1 & 0 & 0 & 0 & 0 & 0 & 0 & 0 & 0 & 0 & 0 & 0 & 0 & 0 & 0 & 0 & 0\\ 0 & 0 & 1 & 1 & -1 & -1 & 0 & 0 & 0 & 0 & 0 & 0 & 0 & 0 & 0 & 0 & 0 & 0 & 0\\ 0 & 0 & 0 & -1 & 1 & 0 & 0 & 0 & 0 & 0 & 0 & 0 & 0 & 0 & 0 & 0 & 0 & 0 & 0\\ 0 & 0 & 0 & 0 & 0 & 1 & 1 & 1 & -1 & -1 & -1 & -1 & 0 & 0 & 0 & 0 & 0 & 0 & 0\\ 0 & 0 & 0 & 0 & 0 & 0 & 0 & 0 & 0 & 0 & 0 & 1 & -1 & 0 & 0 & 0 & 0 & 0 & 0\\ 0 & 0 & 0 & 0 & 0 & 0 & -1 & -1 & 1 & 0 & 0 & 0 & 0 & 0 & 0 & 0 & 0 & 0 & 0\\ 0 & 0 & 0 & 0 & 0 & 0 & 0 & 0 & 0 & 0 & 1 & 0 & 0 & 0 & 0 & 0 & 0 & 0 & 0\\ 0 & 0 & 0 & 0 & 0 & 0 & 0 & -1 & 0 & 1 & 0 & 0 & 0 & -1 & -1 & 0 & 0 & 0 & 0\\ 0 & 0 & 0 & 0 & 0 & 0 & 0 & 1 & 0 & 0 & 0 & 0 & 0 & 0 & 1 & -1 & 0 & 0 & 0\\ 0 & 0 & 0 & 0 & 0 & 0 & 0 & 0 & 0 & 0 & 0 & 0 & 0 & 1 & 1 & 0 & 1 & -1 & -1\\ 0 & 0 & 0 & 0 & 0 & 0 & 0 & 0 & 0 & 0 & 0 & 0 & 0 & 0 & -1 & 0 & -1 & -1 & 0\\ 0 & 0 & 0 & 0 & 0 & 0 & 0 & 0 & 0 & 0 & 0 & 0 & 0 & 0 & 0 & 1 & 0 & 0 & 0\\ 0 & 0 & 0 & 0 & 0 & 1 & 0 & 0 & 0 & 0 & 0 & 0 & 0 & 0 & 0 & 1 & 0 & 0 & 0\\ 0 & 0 & 0 & 0 & 0 & 0 & 0 & 0 & 0 & 0 & 0 & 0 & 0 & 0 & 0 & 0 & 0 & 0 & 1 \end{array}\right)\left(\begin{array}{c} r_{1}\\ r_{2}\\ r_{3}\\ r_{4}\\ r_{5}\\ r_{6}\\ r_{7}\\ r_{8}\\ r_{9}\\ r_{10}\\ r_{11}\\ r_{12}\\ r_{13}\\ r_{14}\\ r_{15}\\ r_{16}\\ r_{17}\\ r_{18}\\ r_{19} \end{array}\right)X$$

### 2.4 Parameter estimation and sensitivity analysis

The model parameters were fitted using the least squares method, with the model’s balance equations serving as constraints. This led to the following optimization problem:

$$\begin{array}{ll} & \underset{\theta }{\min}\frac{1}{2}\underset{t\in t_{\mathrm{meas}}}{\sum }e{\left(t\right)}^{T}W\;e\left(t\right)\\ & s.t.\\ & \;\frac{dC}{dt}=v\;r\;X\\ & \;\theta_{\min}\leq \theta \leq \theta_{\max} \end{array}$$ (27)

where $e\left(t\right)={\left[e_{1},\ldots ,e_{m}\right]}^{T}$ is the discrepancy vector at sampling time *t* formed by the *m* components measured in the experiments, such that $e_{i}\left(t\right)=C_{i}^{\mathrm{pred}}\left(t\right)-C_{i}^{\mathrm{meas}}\left(t\right)$ corresponds to the difference between the predicted and measured concentration of *i* at time *t*. **W** is a scaling matrix that ensures all discrepancies have the same order of magnitude (Dennis and Schnabel, 1996), and ***θ*** represents the set of parameters belonging to the kinetic expressions of the reactions shown in Eqs. 7–25. The experimental values used to fit the model were taken from Shinto et al. (2007) and Al-Shorgani et al. (2018).

A parametric sensitivity analysis was performed to identify the parameters of the biochemical reactions that have the greatest effect on ABE production. The local dynamic sensitivity of the concentrations obtained from the model with respect to the kinetic parameters is quantified using the expression:

$$S_{i,\theta_{j}}\left(\tau \right)=\frac{C_{i}\left(\theta^{*},\tau \right)-C_{i}\left(\theta ,\tau \right)}{C_{i}\left(\theta ,\tau \right)};\;C_{i}\left(\theta ,\tau \right)\neq 0$$ (28)

where *τ* is the time at which the sensitivity is calculated, *i* identifies the component of interest, ***θ*** represents the fitted parameters using the least squares method, Eq. 27, and ***θ*** * is the set of parameters in which the value of the *j*-th parameter of ***θ*** has been perturbed, i.e., $\theta^{*}\;=\;{\left[\theta_{1},\theta_{2},\ldots ,\theta_{j}+\Delta \theta_{j},\ldots ,\theta_{n}\right]}^{T}$ .

The routines for solving the model, Eq. 26, parameter estimation, Eq. 27, and parametric sensitivity analysis, Eq. 28, were implemented in MATLAB using the functions *ode15s* for integrating the system of differential equations and *fmincon* with the Levenberg-Marquardt method for parameter estimation.

## 3 Results and discussion

### 3.1 Model fitting

Parameter identification was performed to fit the model to the experimental data set reported by Shinto et al. (2007), obtained from batch fermentation of *C. saccharoperbutylacetonicum* N1-4. The initial conditions used were: glucose (70.6 mM), biomass (0.20 mM), acetate (40.12 mM), butyrate (2.12 mM), acetone (2.58 mM), and butanol (4.46 mM). The addition of exogenous acetate to the culture medium (Shinto et al., 2007) is explained by its observed effects on metabolic fluxes, leading to a significant increase in acetone and butanol production (Gao et al., 2015).

The proposed model structure was validated by performing parameter identification using the experimental data reported by Al-Shorgani et al. (2018) from a batch fermentation of *C. acetobutylicum* in a 5 L bioreactor with an initial glucose concentration of 50 g L^−1^ (277.78 mM).

**FIGURE 2:**
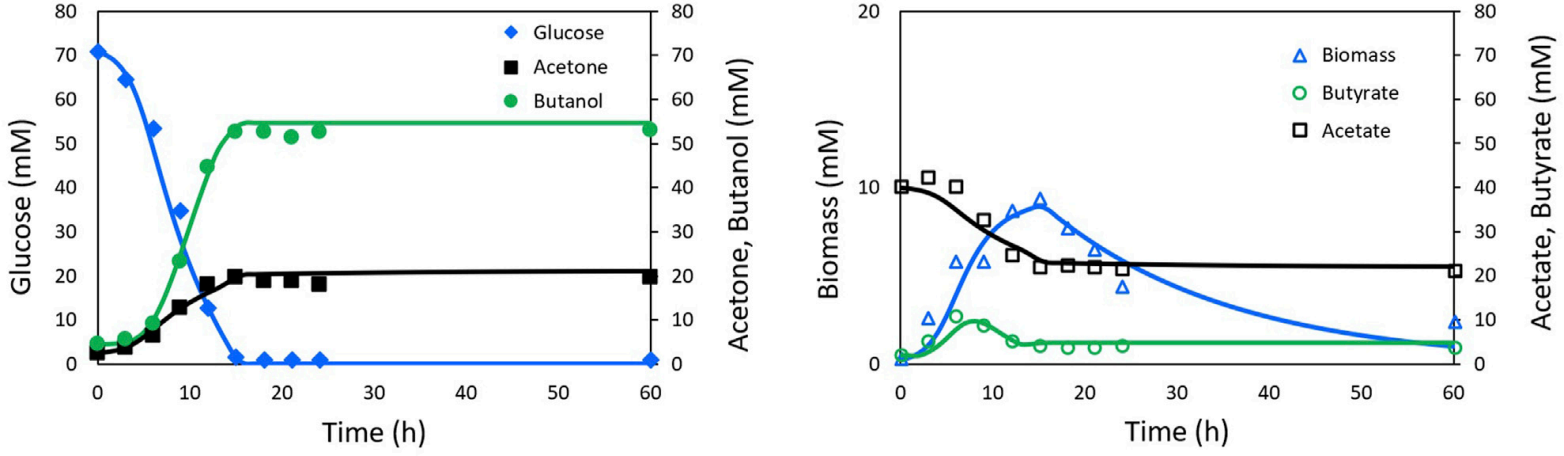
Experimental Shinto data (markers) and simulation (lines) of ABE fermentation with NADH model.

**FIGURE 3:**
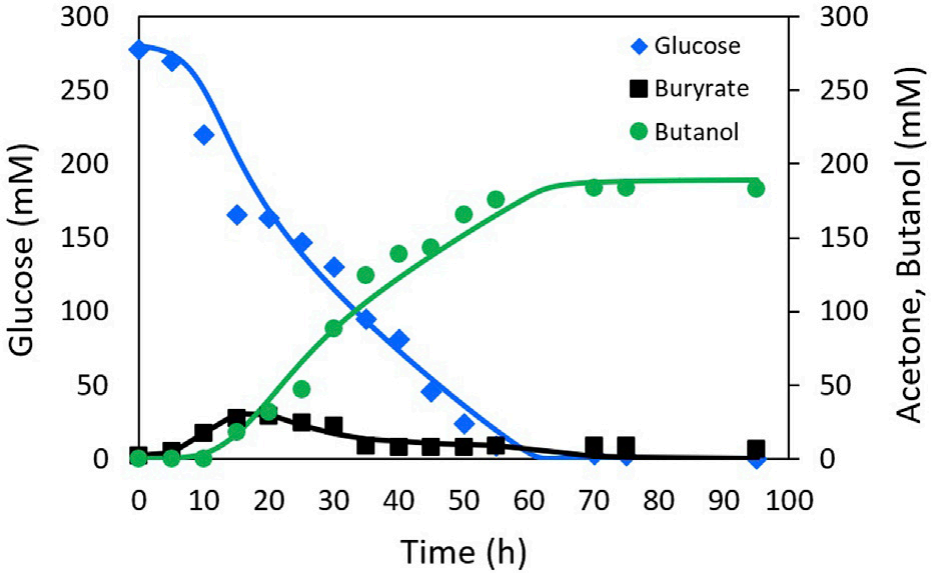
Experimental Al-Shorgani data (markers) and simulation (lines) of ABE fermentation with NADH model.

The fitting of the model to the experimental data from Shinto et al. (2007) and Al-Shorgani et al. (2018) is presented in Figures 2, 3, respectively. The Pearson’s correlation coefficient between each measured and simulated variable is presented in Table 1. For all correlations, a probability value *p* < 0.005 was obtained, indicating no significant differences between the model predictions and the experimental values.

**TABLE 1:** Pearson’s correlation coefficients between experimental data and simulated results.

Model	Glucose	Biomass	Acetate	Butyrate	Acetone	Butanol
NADH model fitted to Shinto	0.997	0.943	0.979	0.819	0.985	0.998
NADH model fitted to Al-Shorgani	0.992	—	—	0.916	—	0.990
Shinto’s Model II	0.988	0.888	0.874	0.863	0.990	0.969
Shinto’s Model III	0.989	0.888	0.983	0.918	0.991	0.986

The results of the model fitting show that a good representation can be achieved without relying on the on-off functions widely used in this type of model, thanks to the inclusion of functions closely related to solvent production, such as the NADH cofactor.

**FIGURE 4:**
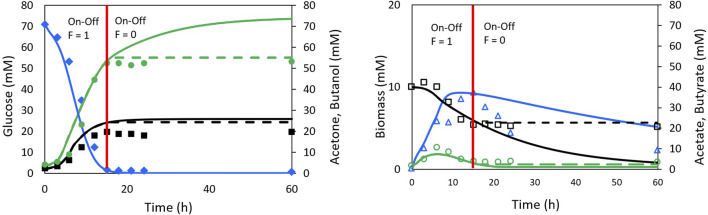
Experimental data and simulation with Shinto II model (continuous line) and Shinto III model (dash line) for ABE fermentation. Data taken from Shinto et al. (2007). Glucose (♢ full blue), Acetone (□ full black), Butanol (○ full green), Biomass (△ empty blue), Acetate (□ empty black) and Butyrate (○ empty green). For Glucose and biomass the lines of the two models are overlapping. The red line represents the moment at which the on-off function in the Shinto III model generates the change in the model behaviour.

The so-called Model II (Shinto et al., 2007), which does not include the on-off function, showed poor description of the ABE process behavior. Model III (Shinto et al., 2007), includes the on-off function to sets reaction rates to zero when the glucose concentration reaches depletion. Although Model III does not explain the underlying phenomena in the biological process, it achieves a better fit than Model II. Figure 4 presents the results obtained by Shinto et al. (2007) with Models II and III, illustrating the degree of fit achieved. Table 1 shows the correlation coefficients for Shinto’s Models II and III compared to those obtained in this work.

Based on the correlation coefficient values, it can be concluded that the proposed model in this work provides a better fit for the ABE fermentation in terms of glucose, biomass, and butanol profiles. An equivalent fit was obtained for the acetate and acetone profiles, and a slightly lower fit was obtained for the butyrate profile compared to Shinto’s Model III. These results highlight the importance of including the NADH cofactor in the kinetic model, as it has a significant influence on electron transport, metabolic flux redistribution, and energy production. Since 4 mol of NADH are consumed for the production of 1 mol of butanol, it is expected that NADH limits the formation of butanol. Therefore, recent studies have focused on increasing the intracellular NADH concentration through genetic engineering of *Clostridium* (Cheng et al., 2019), exogenous addition of NADH or its precursors to the culture medium (Li et al., 2014; Mogollón et al., 2023), or the implementation of electrofermentation strategies (Guerrero et al., 2022; Mogollón et al., 2023).

The values of the parameters obtained through the minimization of the objective function, Eq. 27, are presented in Table 2. Some key parameters in the model, such as the specific glucose consumption rate and the growth rate, show results of the same order of magnitude as those reported by other authors, ranging from 1.62 to 6.79 h^−1^ and from 0.126 to 2.43 h^−1^, respectively (Raganati et al., 2015; Buehler and Mesbah, 2016). However, it should be noted that these parameters are dependent on the cultivation conditions and the strain used, making direct comparisons challenging.

**TABLE 2:** NADH model parameters. NADH model fit A fits experimental data reported by Shinto et al. (2007). NADH model fit B adjusts the experimental data reported by Al-Shorgani et al. (2018).

NADH model fit A					NADH model fit B			
	*v* (*h* ^-1^)	*k*_*A*_ (*mM*)	*k*_*B*_ (*mM*)	*k*_*C*_ (*mM*)	*v* (*h* ^-1^)	*k*_*A*_ (*mM*)	*k*_*B*_ (*mM*)	*k*_*C*_ (*mM*)
*r*_1_	9.89	11.54	89.50	2.56	7.30	42.40	62.98	5.11
*r*_2_	41.10	4.0E-04			44.84	3.20E-5		
*r*_3_	148.27	4.91E-2			144.84	16.93		
*r*_4_	14.23	154.19			24.61	172.78		
*r*_5_	6.22E-2	494.70			2.06E-3	502.49		
*r*_6_	166.00	0.31			178.808	2.53		
*r*_7_	5.40E-3	111.61			1.14E-4	92.65		
*r*_8_	144.91	0.85	12.77		106.84	1.51E-2	19.88	
*r*_9_	2.18	85.45			5.96	65.19		
*r*_10_	100.23	3.63E-1			83.61	0.49		
*r*_11_	7.63E-1	46.31	26.27		15.95	37.62	46.45	
*r*_12_	6.35	0.11	144.58		16.16	1.05	155.71	
*r*_13_		5.06E-2				5.77E-4		
*r*_14_	44.43	7.42E-1	2.40		9.00	4.51	1.29	
*r*_15_	3.55	4.30	53.62		91.37	26.79	46.78	
*r*_16_	44.76	2.43E-1		2.56		1.09		
*r*_17_	91.59	2.85	2.36		15.60	23.78	14.96	
*r*_18_	4.84	12.66		2.56		17.35		
*r*_19_	26.27	6.0E-1	28.45	105.51	14.61	1.82	32.49	81.06

### 3.2 Parametric sensitivity

**FIGURE 5:**
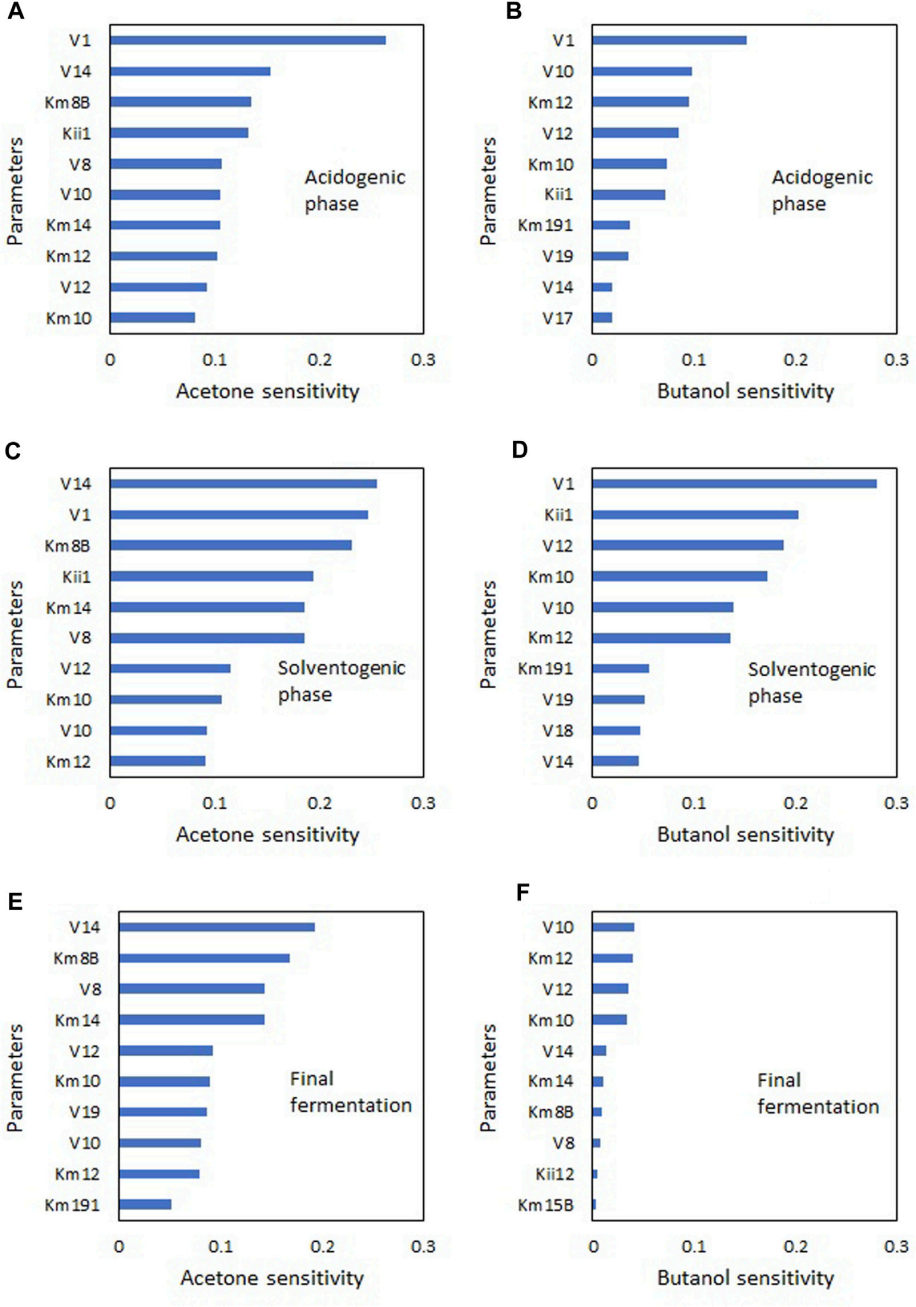
Parametric sensitivity (±20%) in NADH ABE model at different stages of ABE fermentation. **(A)** Acetone-acidogenic phase, **(B)** Butanol-acidogenic phase, **(C)** Acetone-solventogenic phase, **(D)** Butanol-solventogenic phase, **(E)** Acetone-final fermentation, **(F)** Butanol-final fermentation.

A sensitivity analysis was carried out to identify the parameters with the greatest influence on solvent production (acetone and butanol). Therefore, the parameter values were varied by ± 5% to ±50% around the base values in Table 2. The sensitivity of the parameters for a ±20% variation is shown in Figure 5 for the 10 most sensitive parameters. Due to the nonlinearity of the model, parameter sensitivity varies over time. Therefore, the sensitivities in Figure 5 are presented for three time points during the fermentation process: 5 h, corresponding to the acidogenic phase (Figures 5A, B); 15 h, corresponding to the solventogenic phase (Figures 5C, D); and 60 h, the end of the process (Figures 5E, F).

The most sensitive parameter in the acidogenic phase was the specific glucose consumption rate (*V*_1_), which is consistent with the direct relationship between the production of acetic and butyric acids and glucose consumption. In this phase, a 20% variation in the base parameter value generates higher sensitivity towards acetone (26%) than to butanol (15%).

In the solventogenic phase, the most sensitive parameters were *V*_14_ for acetone (26%) and *V*_10_ for butanol (28%). These results indicate that the active pathway in the ABE metabolism corresponds to the glucose → pyruvate → acetyl-CoA → butyryl-CoA axis. Although acid re-assimilation for solvent production is important in this phase, a direct pathway for butanol production called the “*hot channel*” has been described, which would require significant metabolic activity in this channel. In recent research, the hot channel pathway has also been identified as a significant route for butanol production without the need for acid accumulation (Jang et al., 2023; Liu et al., 2023; Mogollón et al., 2023). The high sensitivity of the parameter *V*_8_ to acetone also suggests that the re-assimilation pathway known as the “*cold channel*” is active. Parameter sensitivity at the end of the process shows low activity of the “*hot channel*”, while the activity of the “*cold channel*” towards acetone is still high due to the presence of acetate in the medium.

The importance of parameter analysis at different time points in the process lies in the possibility of determining not only the parameters that affect the final product values but also increasing their productivities. For example, the parameter *V*_12_, which corresponds to the specific growth rate, has a significant effect on butanol production in the solventogenic phase but little significance in the final phase of the culture. This indicates that it would be possible to achieve higher butanol productivity by modifying this parameter while maintaining the culture in steady-state in the solventogenic phase. This parameter is particularly important in continuous culture processes (Buehler and Mesbah, 2016; Díaz and Willis, 2018) demonstrating that under steady-state conditions butanol productivity increases with increasing dilution rates.

**FIGURE 6:**
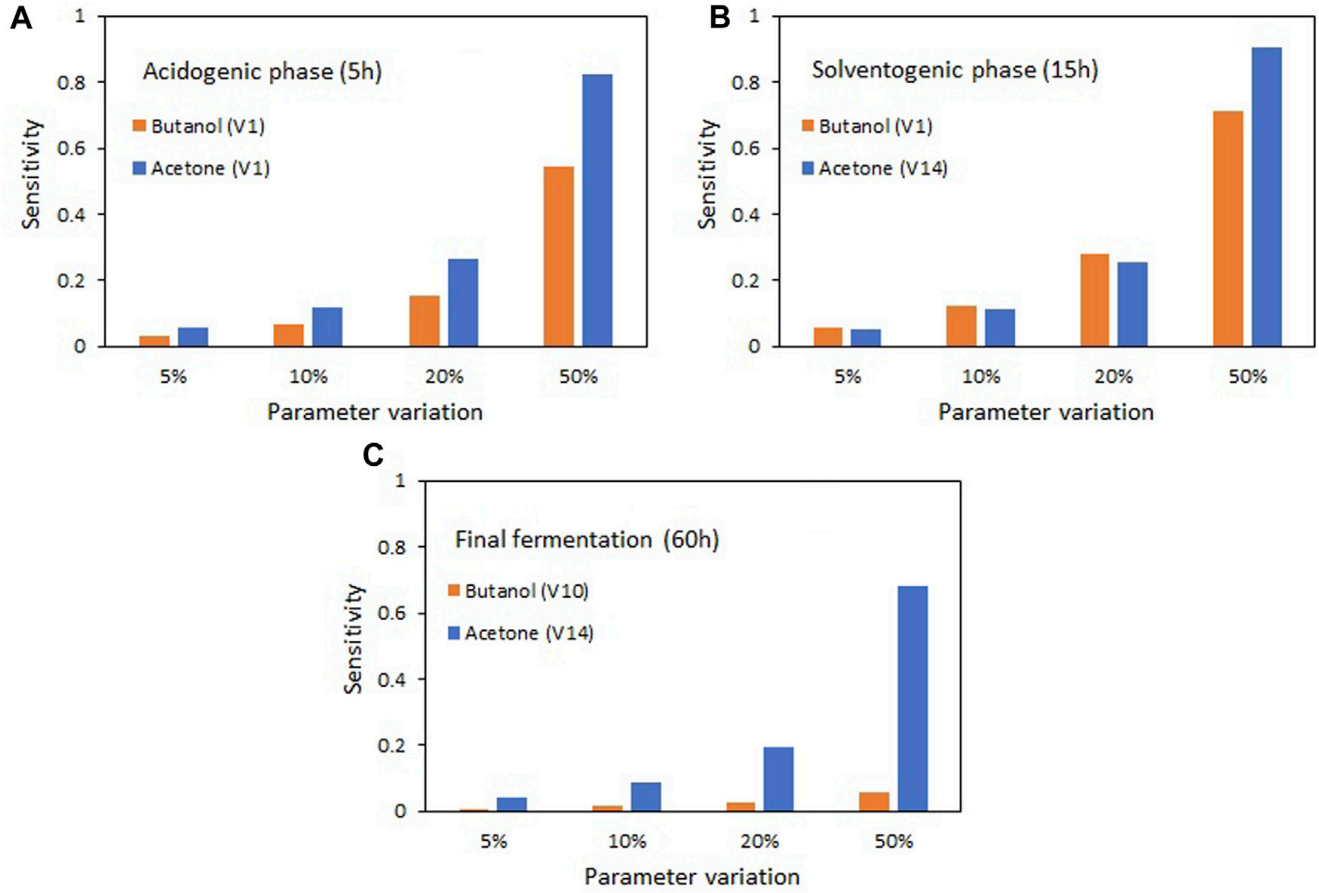
Effect of changes in the most sensitive parameters on the production of acetone and butanol for the different stages of the ABE fermentation. **(A)** Acidogenic phase (5 h), **(B)** Solventogenic phase (15 h), **(C)** Fermentation end time (60 h).

Figure 6 shows how the variation in parameter values between 5% and 50% affects the results of the state variables acetone and butanol. As the variation of parameters *V*_1_ (related to the maximum glucose consumption rate) and *V*_14_ (related to the maximum transformation rate of acetoacetyl CoA to butyryl CoA) increases, there is a noticeable rise in the production of acetone and butanol during the acidogenic (Figure 6A) and solventogenic (Figure 6B) phases. This observation suggests that enhancing the activity of enzymes involved in these reaction stages will subsequently lead to an improvement in solvent production. However, in the final fermentation stage (Figure 6C), when glucose is depleted, the increase in the most sensitive parameter (*V*_10_) has a negligible impact on butanol production. Conversely, for acetone, it remains possible to achieve greater production increments by elevating the activity of the enzyme associated with parameter *V*_14_. At this stage, the most significant parameters are those involved in acid re-assimilation, although their effect is much smaller than that observed in the acidogenic and solventogenic phases.

The high sensitivity of parameters *V*_1_, *V*_10_, and *V*_14_ towards acetone and butanol production highlights the key role of enzymes associated with those compounds in ABE metabolism. Their overexpression has been considered for improving solvent production. Parameter *V*_1_ is associated with the phosphoenol pyruvate-dependent phosphotransferase system (PTS), where substrate consumption and phosphorylation occur simultaneously (Mitchell, 2015). Recent studies have examined the expression of the *glcG* gene encoding glucose transport proteins in the PTS system of *C. acetobutylicum* and have found up to a 300% increase in butanol production compared to control strains (Wu et al., 2019). Parameter *V*_10_ is associated with the enzyme thiolase (acetyl-CoA-acetyltransferase), which catalyzes the condensation of two molecules of acetyl-CoA to form one molecule of acetoacetyl-CoA. Overexpression studies of the *thlA* gene in *C. acetobutylicum* have shown increases in butanol production ranging from 18% to 64% (Mann and Lütke-Eversloh, 2013; Kim et al., 2015). Similarly, parameter *V*_14_ is associated with the enzymes beta-hydroxybutyryl-CoA dehydrogenase (BHBD), enoyl-CoA hydratase (crotonase) (ECH), and butyryl-CoA dehydrogenase (BCD), involved in the reduction of acetoacetyl-CoA to butyryl-CoA. Overexpression of the genes encoding these enzymes has led to an 8% increase in butanol in *C. saccharoperbutylacetonicum* (Tian et al., 2019) and a 2.2-fold increase in *C. thermocellum* (Hussain et al., 2023).

The studies described are in agreement with the results obtained with our model, which identifies key intervention points for metabolic engineering strategies to enhance fermentation efficiency.

### 3.3 Effect of process variables on ABE production

**FIGURE 7:**
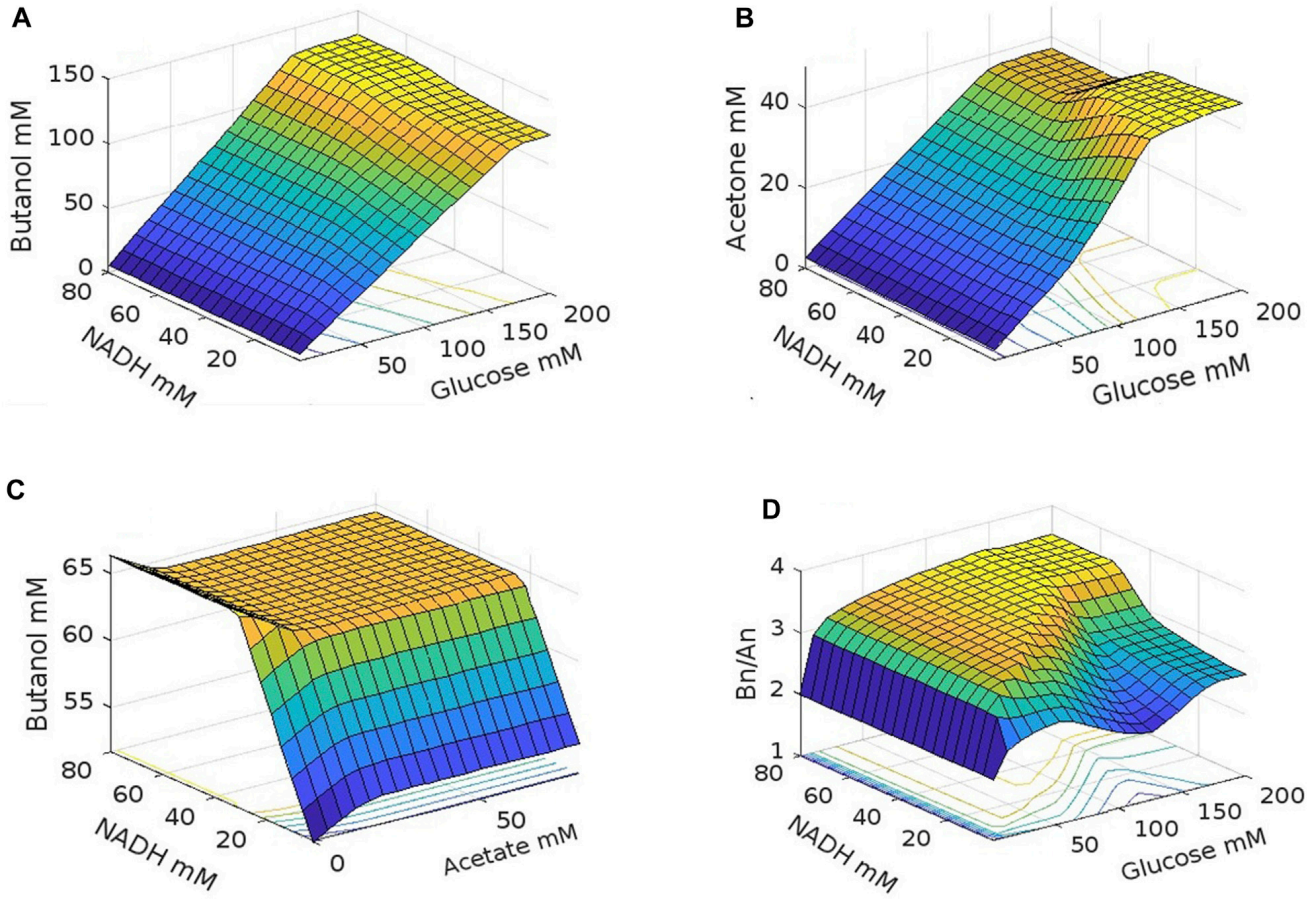
NADH, glucose and acetate effect on ABE fermentation. **(A)** Glucose, NADH effect on butanol, **(B)** Glucose, NADH effect on acetate, **(C)** NADH, acetate effect on butanol, **(D)** Glucose, NADH effect on butanol acetate selectivity.

Based on the proposed mathematical model, simulations were performed to determine the effects of NADH, glucose, and acetate concentrations on ABE fermentation. Figure 7 shows the results for butanol and acetone production, and the butanol/acetone selectivity (Bn/An). It can be observed (Figures 7A, B), that both butanol and acetone production increase simultaneously with increasing glucose concentration up to maximum values of 160 mM for butanol and 140 mM for acetone. Beyond these concentrations, further increases in glucose do not cause any significant increase in acetone and butanol production. The relationship between glucose concentration and butanol production is linear. At very low glucose concentrations (carbon limitation) solvent production is low, and acids are accumulated. Conversely, at high concentrations of glucose, substrate inhibition occurs, a phenomenon widely reported in ABE fermentation (Ezeji et al., 2004; Al-Shorgani et al., 2011; Buehler and Mesbah, 2016).

Regarding NADH, butanol concentration increases with increasing NADH concentration, up to a maximum value beyond which butanol production is not affected by further increases in NADH concentration (Figure 7A). However, the demand for NADH increases with glucose concentration. For instance, at a glucose concentration of 67 mM, the maximum butanol production (60 mM) is obtained at 18 mM NADH, while at a glucose concentration of 86 mM, the maximum butanol concentration (77 mM) is reached at 27 mM NADH. This can be explained by the fact that *C. acetobutilicum* can produce 1 mol of butanol per mole of glucose consumed. However, in the EMP pathway, 1 mol of glucose yields 2 mol of NADH, while the production of 1 mol of butanol requires 4 mol of NADH and 1 mol of NADPH (see Figure 1). Additionally, the ferredoxin cycle allows for the generation of additional NADH through the decarboxylation of pyruvate to acetyl-CoA, however, a portion of this NADH is converted into NADPH in the same cycle (Wang et al., 2010; Foulquier et al., 2022). This means that the biosynthesis of butanol is typically limited by the availability of NADH, resulting in low concentration, low yield, and low productivity (Du et al., 2015). These results indicate that a higher glucose consumption rate leads to a higher demand for NADH.

As the butanol concentration increases with increasing NADH concentration, there is a decrease in acetone production (Figure 7B), indicating that the carbon required for butanol production is obtained at the expense of reduced acetone production. Since butanol production is used by the cell for NAD^+^ regeneration, an imbalance in the redox state is generated with increased NADH concentration, which is compensated by an increased production of butanol. This mechanism, evident through the proposed mathematical model in this work, has also been recently identified experimentally, enabling the improvement of butanol production by inducing a redox imbalance in the cell through the application of exogenous NADH or precursors such as nicotinic acid or other redox mediators carrying electrons such as methyl viologen (Jang et al., 2014; Li et al., 2014; Du et al., 2015; Liao et al., 2019; Mogollón et al., 2023), or by varying the extracellular redox potential through the application of an external voltage, which can induce a change in the intracellular redox potential. This latter strategy is considered the basis for the development of electrofermentation processes (Guerrero et al., 2022; Mogollón et al., 2023).

In Figure 7C, the effect of NADH on butanol production is more visible. The data also shows that increasing the concentration of exogenous acetate in the medium up to approximately 20 mM also increases butanol production. This effect remains until NADH concentrations reach approximately 30 mM. At higher NADH values, the presence of exogenous acetate has a negative effect on butanol production, while acetate concentrations above 20 mM at low NADH values have no effect on butanol production. The effect of acetate has been extensively studied, and it has been found that other organic acids such as butyrate, valerate, propionate, among others, have a similar effect (Grupe and Gottschalk, 1992).

During ABE fermentation, the pH of the culture decreases, but the intracellular pH decreases less than the extracellular pH. It is believed that the presence of weak organic acids such as acetate can induce early the shift from the acidogenic phase to the solventogenic phase due to their ease of entry into the cell as undissociated acid. Once inside the cell, the acid dissociates and reduces the intracellular pH, leading to a faster decrease in intracellular pH and promoting the initiation of solvent production (Grupe and Gottschalk, 1992). Acetate and butyrate have also been identified as inducers in butanol synthesis, acting as environmental signals that reduce “cell degeneration,” which refers to the loss of the solventogenic operon (*sol*) containing the solventogenic genes (*ctf*A, *ctf*B, and *adhE/aad*) (Chen and Blaschek, 1999).

The results of the model show that the increase in butanol production is induced by an increase in NADH concentration, resulting in a reduction in acetone production. Figure 7D depicts the combined effect of acetone and butanol production (selectivity) towards butanol as a function of NADH and glucose concentration. It can be observed that increasing the concentration of glucose at low concentrations linearly increases selectivity, regardless of the NADH concentration. From 10 mM glucose and physiological concentrations of NADH, which range from 0.039 to 8.49 mM (Zhou et al., 2011; Zhang et al., 2014), selectivity remains relatively constant, indicating that both acetone and butanol are produced at the same rate. At 50 mM glucose, a reduction in selectivity towards butanol is observed, corresponding to an increase in acetone production (Figure 7B), while butanol is maintained at a constant production rate (Figure 7A). At around 126 mM glucose, acetone production stabilizes while butanol continues to grow, resulting in an increased selectivity towards butanol. From 168 mM glucose, selectivity stabilizes because both acetone and butanol production reach a stable state. Under these physiological NADH concentration conditions, a selectivity minimum of 2.1 is observed at a glucose concentration of 126 mM. Increasing the NADH concentration above its physiological level increases selectivity towards butanol for any glucose concentration, reaching a maximum selectivity of 3.6 at NADH concentrations above 55 mM and a glucose concentration of 126 mM. Comparable outcomes have been achieved through recently proposed, more complex models based on cell-free systems. In these models, the highest concentrations of butanol are reached at 50 mM NADH concentrations (Martin et al., 2023).

The results obtained with the proposed mathematical model align with experimental observations made by various authors and emphasize the importance of NADH as a redox agent in the cellular metabolism of *Clostridium*. This model not only enables the analysis of cellular metabolism through process simulation but also facilitates the identification of suitable operating conditions to increase the productivity and selectivity of solvents in ABE fermentation, particularly butanol.

## 4 Conclusion

In this contribution, a new mathematical model was developed for ABE solvent production using mass balance equations and biochemically-based kinetic expressions, replacing some artificial functions used in previous models. The structure of the model allowed for an adequate representation of the experimental values reported in other studies, with performance equal to, or better than models developed in previous works. The inclusion of NADH was crucial in eliminating the need for artificial variables.

The parametric sensitivity analysis determined that the metabolic reactions for substrate consumption and butyryl-CoA production from acetyl-CoA are the most significant within *Clostridium* metabolism. This supports the metabolic engineering strategies to improve butanol production by manipulation of those reactions. Furthermore, the analysis of ABE fermentation through process simulation shows that an increase in intracellular NADH concentration significantly enhances butanol production and selectivity over acetone.

The inclusion of NADH effect on the cellular metabolism model provides a basis for constructing models describing the dynamics of bioprocesses mediated by electrochemical processes. This opens up possibilities for inducing an increase in intracellular NADH concentration by modifying the extracellular redox potential through the application of an external electric potential, aiming to improve yields compared to traditional fermentations.

## Figures and Tables

**Table:** 

**ABE**	Acetone-butanol-ethanol
**NADH**	Nicotinamide Adenine Dinucleotide
**ATP**	Adenosine triphosphate
**[*G*]**	Glucose concentration, mM
**[*F*6*P*]**	Fructose 6-P concentration, mM
**[*G*3*P*]**	Glyceraldehyde 3-P concentration, mM
**[*Lac*]**	Lactate concentration, mM
**[*Pyr*]**	Pyruvate concentration, mM
**[*Ac*]**	Acetate concentration, mM
**[*ACoA*]**	Acetyl-CoA concentration, mM
**[*X*]**	Biomass concentration, mM
**[*EtOH*]**	Ethanol concentration,mM
**[*AACoA*]**	Acetoacetyl-CoA concentration, mM
**[*AcAc*]**	Acetoacetate concentration, mM
**[*Buty*]**	Butyrate concentration, mM
**[*BCoA*]**	Butyryl-CoA concentration, mM
**[*An*]**	Acetone concentration, mM
**[*BuOH*]**	Butanol concentration, mM
**[*NADH*]**	Nicotinamide Adenine Dinucleotide concentration, mM

## Data Availability

The original contributions presented in the study are included in the article/Supplementary Material, further inquiries can be directed to the corresponding author.
